# Honeybees Collecting Latex From Breadfruit (*Artocarpus altilis*) in Puerto Rico Reveal a Novel Interaction With Broad Eco‐Evolutionary Implications

**DOI:** 10.1002/ece3.72647

**Published:** 2025-12-05

**Authors:** Luis Y. Santiago‐Rosario, Juan A. Santiago‐Rosario, Emilie C. Snell‐Rood

**Affiliations:** ^1^ Department of Ecology, Evolution and Behavior University of Minnesota St. Paul Minnesota USA; ^2^ Centro de Genómica, Ecología y Medio Ambiente (GEMA) Universidad Mayor Santiago Chile; ^3^ Department of Natural Sciences and Mathematics Inter American University of Puerto Rico Bayamón Puerto Rico USA

**Keywords:** behavioral plasticity, Caribbean, ecological interactions, nonnative species, plant chemistry, plant secondary compounds

## Abstract

We document honeybees collecting latex from breadfruit in Puerto Rico, a rare interaction that reveals how plant chemical diversity can shape bee foraging behavior, plant–insect dynamics, and evolutionary trajectories highlighting the role of nonnative species in driving eco‐evolutionary change in tropical ecosystems.

## Graphical abstract

This article showcases a unique natural history observation of honeybees collecting latex from the breadfruit tree ( *Artocarpus altilis* ), both nonnative species in Puerto Rico. This represents a unique interaction and here we explore how this behavior may come about in novel ecosystems.

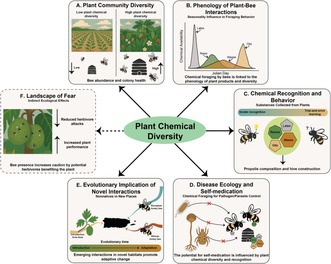

## 1 Introduction and Observation

The spatial structure of plant chemistry, including both elemental composition and secondary metabolites, plays a fundamental role in shaping the ecology and evolutionary trajectories of herbivores (Hunter 2016). Plants exhibit variation in their chemical profiles, driven by geographic, biogeochemical, and/or phylogenetic factors (Santiago‐Rosario et al. 2022; Watanabe et al. 2007). This variation results in heterogeneous environments that animals must navigate to fulfill their nutritional requirements (Hunter 2016; Sterner and Elser 2002), which is particularly pronounced in tropical regions due to the high plant biodiversity. In response, animals often develop specific foraging strategies that enable them to tolerate/exploit chemical extremes—whether coping with deficiencies or tolerating high concentrations of compounds or elements (Provenza et al. 2007; Santiago‐Rosario et al. 2023; Zangerl and Berenbaum 1993).

Insects exploit a wide variety of chemical niches and interact with plants in diverse ways, with tropical ecosystems hosting particularly high levels of herbivore specialization (Forister et al. 2015). However, the role of plant chemical variation in shaping insect foraging patterns—and its broader consequences for tropical plant–insect interactions, macroecology, and evolution—remains poorly understood.

Bees (Apidae: Hymenoptera) are among the best‐studied insects, largely for their nectar and pollen foraging and the resulting contributions to pollination and ecosystem functioning. Less recognized, however, is that many bee species also collect other plant exudates, such as resins, latex, and oils, that provide nonnutritional but ecologically important functions (Henske et al. 2025; Murúa 2020; Simone‐Finstrom and Spivak 2010; Vit et al. 2024; Weissmann and Schaefer 2015). Resins, for example, are complex mixtures of terpenoids, phenolic compounds, and fatty acids produced by specialized tissues, often in response to wounding or herbivory (Dell and McComb 1979). Latex, a milky emulsion of alkaloids, proteins, oils, and gums, is secreted by laticifers distributed across different plant organs (Ramos et al. 2019). Honeybees ( *Apis mellifera* ) and several stingless bees gather resins to produce propolis which is considered a multifunctional material that strengthens hives, glues combs to substrates, and provides antimicrobial protection (Vit et al. 2024; Wagh 2013). In tropical regions, propolis can contain more than 800 distinct plant‐derived compounds (Vit et al. 2024). Beyond hive construction, bees and other insects may also use such exudates as a form of self‐medication, selectively foraging on bioactive compounds when challenged by pathogens or parasites, improving survival (Abbott 2014). These behaviors highlight the sophisticated ways in which bees exploit plant chemical defenses, extending their ecological role far beyond pollination and nutrition.

Despite the extensive literature on bee foraging behavior, the collection of latex remains poorly documented. Latex production represents a convergent trait that evolved across multiple plant clades (Konno 2011). Chemically, latex is highly variable and contains a suite of bioactive compounds, including toxins and deterrents, that discourage herbivory through physical and chemical defense (Agrawal and Konno 2009; Ramos et al. 2019). Although such properties are generally considered antagonistic, direct observations of bees collecting latex suggest that these exudates could also play unexpected roles in mutualistic or beneficial interactions and uses.

**FIGURE 1:**
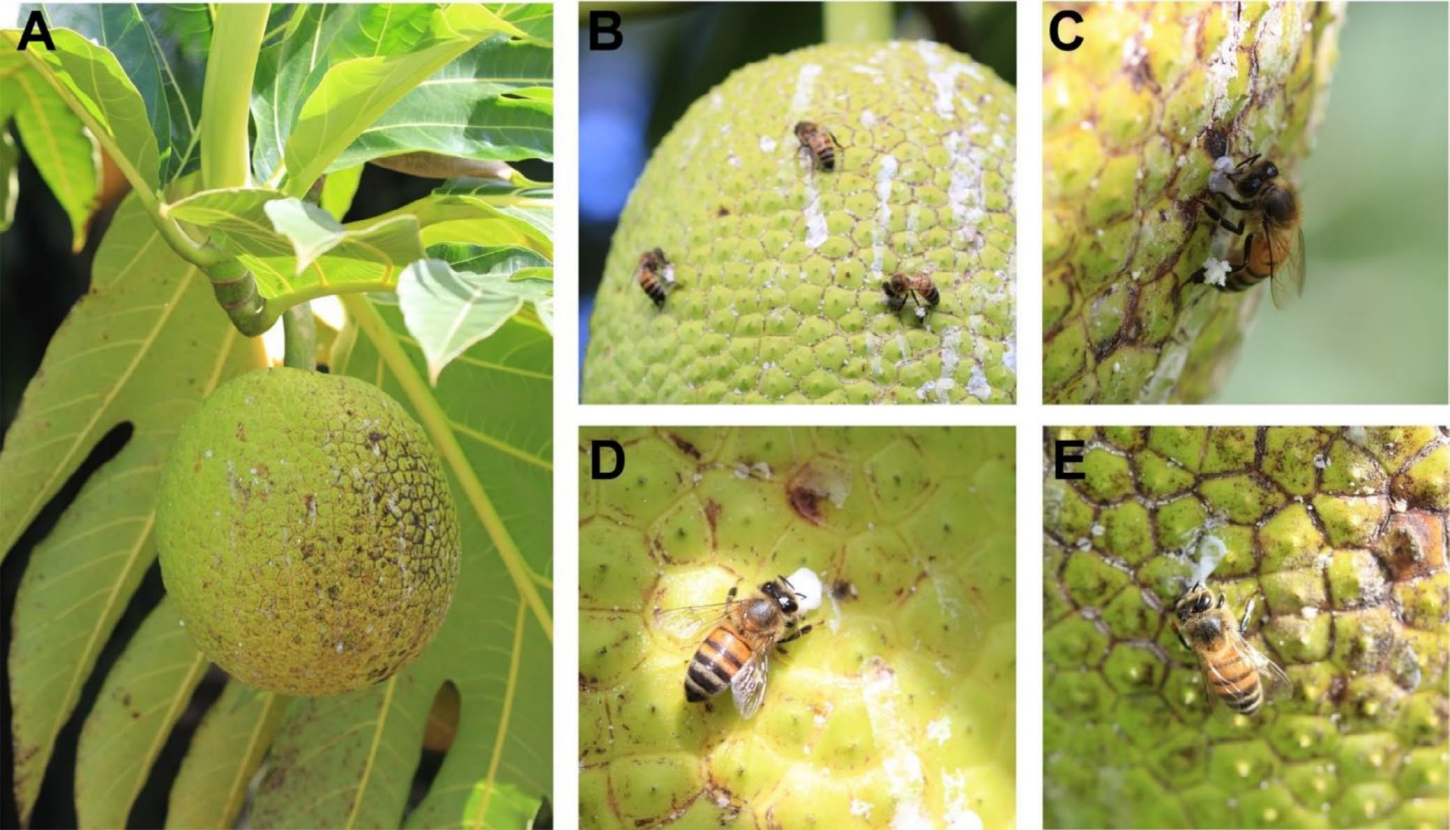
(A) An unripe breadfruit fruit showing latex exudates. (B) Group of honeybees collecting latex. (C) An individual honeybee collecting latex on the unripe breadfruit. Here, it can be observed that the bee is extracting the latex from the breadfruit and the accumulation of the latex in the corbiculae. (D, E) Honeybee manipulating fresh latex exuding from a fresh wound on the breadfruit.

In December 2024, we observed groups of 2–8 honeybees visiting individual low‐hanging fruits across six breadfruit trees, *Artocarpus altilis* (Moraceae: Figure 1A) in Ciales, Puerto Rico (~600 m elevation). Over a 3‐week period, we recorded this behavior during 11 separate systematic walks (following the same path) conducted between 8:00 AM and 12:00 PM. The habitat is a mixture of old coffee plantation interspersed with some nonnative fruit trees (e.g., *Citrus* [Rutaceae], soursop [ *Annona muricata* : Annonaceae], and guava trees [ *Psidium guajava* : Myrtaceae]) and other native trees and shrubs such as *Inga vera* (Fabaceae), *Miconia* (Melastomataceae), *Tabebuia heterophylla* (Bignoniaceae), among other species. *Artocarpus altilis* , along with other relatives of the genus, is native to Southeast Asia and was introduced to the Caribbean ~230 years ago during the transatlantic slave trade as a crop species (Audi et al. 2023; Williams et al. 2017).

During our observations, the large unripe fruits exuded latex, creating a mosaic pattern of fresh, white latex and older, dried brown‐gray exudate (Figure 1A). Interestingly, we observed bees selectively collecting the new latex produced by the fruits every day (Figure 1B–D). After gathering the latex, the bees manipulated it with their mandibles before depositing it into the corbiculae (pollen baskets) for transport. We did not observe any pollen in the corbiculae—the bees appeared to forage selectively for latex. Latex on the fruit showed patterns of fresh, white exudate intermixed with older, dried brown‐gray latex (Figure 1C,D). No other insects were observed visiting the fruits or exhibiting similar behavior, nor did the honeybees interact with other parts of the tree. Observations conducted outside the fruiting period confirmed that honeybees did not forage on the trees when fruits were absent.

Moreover, following the same protocol, an additional survey conducted by JS‐R in April 2025, when trees are not fruiting, recorded no honeybee foraging.

These findings contribute to a growing but understudied body of evidence documenting latex collection by bees and raise important questions about the ecological role and adaptive significance of this behavior, especially for honeybees. In the following section, we propose research directions aimed at exploring how the chemical complexity of latex—often regarded solely as a defensive trait for plants—relates to its potential use by the hive, and whether bees actively select latex based on specific chemical properties. More broadly, we highlight the need to examine how phytochemical diversity shapes foraging strategies, colony performance, and the emergence of novel plant–insect interactions in tropical ecosystems.

## 2 Discussion

Latex collection by bees, though uncommon, has been reported in a limited number of plant taxa, raising questions about its ecological and evolutionary significance. Latex is generally regarded as a defense against herbivores due to its toxicity and stickiness (Agrawal and Konno 2009; Helmus and Dussourd 2005; Konno 2011), yet several bee species forage on it in both temperate and tropical systems. Documented cases include honeybees gathering latex from *Azorina vidalii* (Campanulaceae) in the Azores, Portugal (Weissmann and Schaefer 2015), *Trigona* stingless bees foraging on nonnative *Euphorbia* (Euphorbiaceae) in Brazil (Tietz et al. 2022), and *Tetragonula iridipennis* collecting latex from multiple species in India, with some individuals showing specialization in latex or resin foraging (Layek et al. 2024, 2023).

A potential function of latex collection in eusocial bees is its incorporation into propolis and cerumen, key materials for hive construction and maintenance (Layek et al. 2023; Vit et al. 2024). Latex may contribute both structurally and chemically, strengthening hive architecture while also providing antimicrobial protection. For example, honeybees in Northwest Africa incorporate latex from *Euphorbia officinarum* into propolis, which retains antimicrobial activity from its plant origin (Boutoub et al. 2022). These observations suggest that latex use may contribute to social immunity and colony defense, warranting further investigation into its prevalence across eusocial bees and its potential ecological role in tropical habitats.

Our observations confirm latex collection by honeybees, but its functional role—whether in hive construction, medicinal use, or other contexts—remains unknown. Interestingly, the spatial pattern of latex on the fruit suggests that bees may create small incisions or manipulate the fruit surface during collection, which could facilitate latex flow. While we cannot confirm the function of this behavior, it is reminiscent of latex‐management strategies observed in other taxa, such as monarch butterfly larvae on *Asclepias* leaves (Helmus and Dussourd 2005). This behavior highlights a potential adaptive foraging strategy that warrants further investigation to determine whether it serves specific functional roles, such as enhancing latex collection or accessing particular chemical compounds. Future studies should investigate the chemical composition of *A. altilis* latex, its potential benefits to bees, and its broader implications for plant–insect interactions in the Neotropics.

### 2.1 Emerging Questions in Plant–Bee Chemical Ecology

**FIGURE 2:**
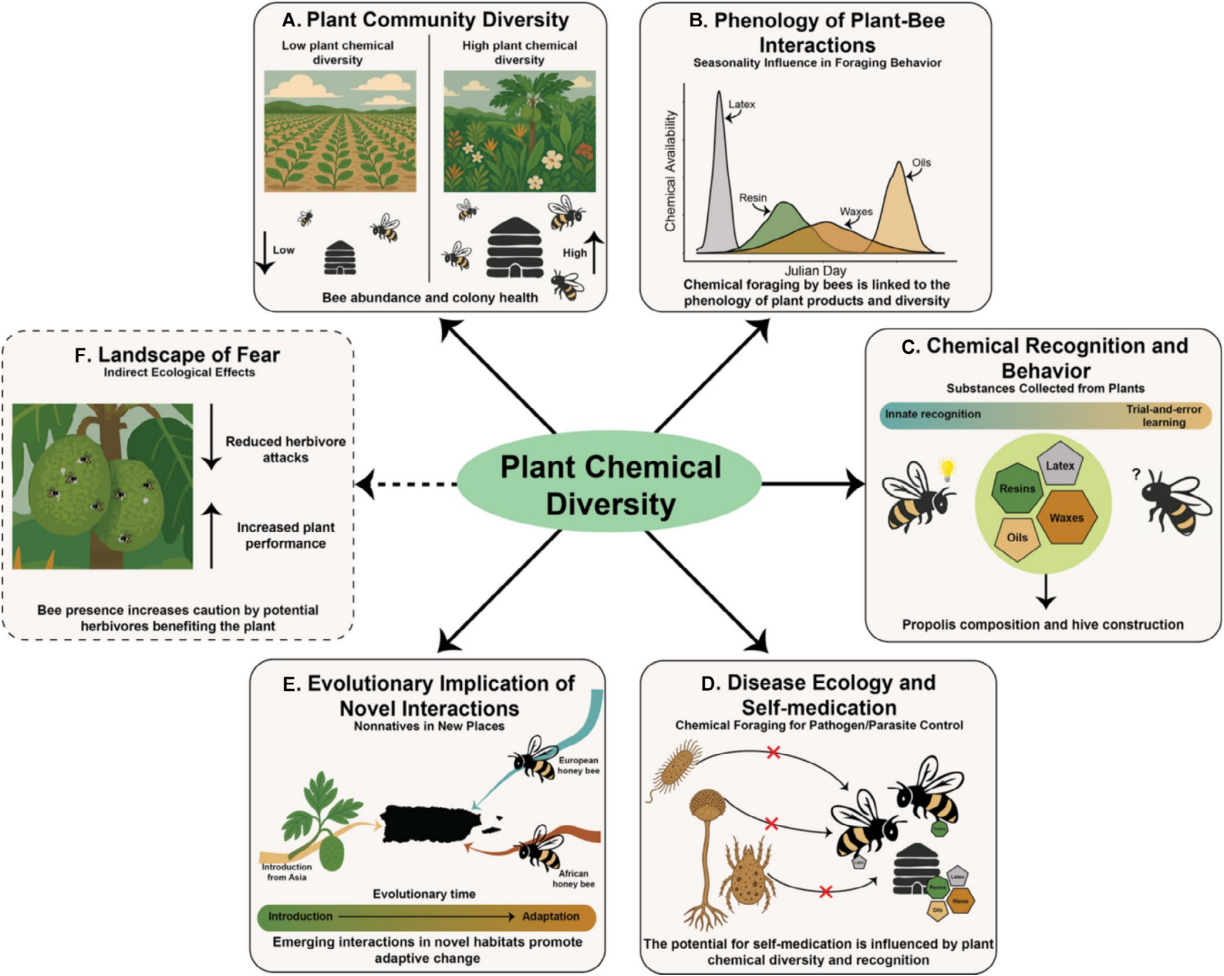
Conceptual diagram illustrating the ecological and evolutionary effects of plant chemical diversity on bee ecology. (A) High chemical diversity supports increased bee abundance and colony health; (B) the chemical production phenology may impact foraging behavior and strategies; (C) chemical recognition and foraging behavior; (D) the impact of plant chemical diversity on disease ecology; (E) the adaptive implications of chemical foraging in new places by nonnative species; and (F) the indirect effects of bee presence in reducing herbivory via a chemically mediated “landscape of fear”.

Our observation of honeybees collecting latex—a behavior rarely documented—opens new avenues for understanding chemically mediated plant–insect interactions in tropical systems. Although based on a single context, this finding highlights the broader need to investigate how plant chemical diversity shapes eusocial bee behavior and ecology. Figure 2 outlines key hypotheses and research directions, including: (A) how plant chemical diversity influences bee colony performance; (B) how the phenology of compound production affects foraging; (C) how bees detect and respond to chemical cues; (D) the role of bioactive compounds in self‐medication; (E) the evolutionary implications of foraging on nonnative resources; and (F) the indirect ecological effects of nonfloral foraging (Figure 2). Although these questions extend beyond the scope of this study, they underscore the potential of chemical ecology to reveal hidden dimensions of plant–bee interactions in the tropics.

#### 2.1.1 Plant Chemical Diversity, Phenology, and Colony Performance

Plant diversity, and the chemical resources it provides, plays a central role in bee foraging and colony health (Jha and Kremen 2013; Mramba 2025). Beyond nectar and pollen, bees exploit resins, oils, and latex produced by plants (Vit et al. 2024). We propose that reduced chemical diversity in monocultures, fragmented habitats, or urban landscapes may restrict access to essential compounds, weakening immune defenses and impairing nest construction (Barroso‐Arévalo et al. 2019; Branchiccela et al. 2019; Drescher et al. 2014; Filipiak et al. 2017). In contrast, diverse tropical systems often provide staggered phenologies of resin‐ and latex‐producing plants, buffering colonies against seasonal gaps. Our latex observation suggests that bees opportunistically exploit compounds as they become available, emphasizing the combined importance of plant chemical diversity and phenological complementarity (Figure 2a,b). Disruption of either could jeopardize access to key resources.

#### 2.1.2 Chemical Recognition and the Role of Medicinal Foraging in Bee Health

To exploit chemically complex resources, bees must detect and evaluate compounds in ways that align with colony needs. This ability relies on both innate sensory biases (toward sweet, sticky, or volatile substances) and learned associations reinforced by social learning (Giurfa 2007; Leadbeater and Dawson 2017). Latex foraging may reflect this combination of evolved preference and behavioral plasticity, enabling bees to incorporate novel resources into their repertoire (Figure 2c). Crucially, recognition mechanisms also link to disease ecology: the selective use of bioactive compounds underpins self‐medication and parasite resistance (Abbott 2014). Latex and resins often contain antimicrobial or antifungal properties that reduce pathogen loads and improve colony health (Simone et al. 2009; Simone‐Finstrom and Spivak 2010; Tao et al. 2016). Thus, the ability to identify and utilize medicinal compounds is a key axis of colony immunity, especially under high parasite pressure in tropical environments (Figure 2d).

#### 2.1.3 Adaptive Consequences of Nonfloral Foraging

Chemically mediated foraging may have long‐term evolutionary implications, particularly in novel or human‐modified environments. In Puerto Rico, for instance, introduced honeybees collect latex from breadfruit, a tree also introduced to the Caribbean (Acevedo‐Gonzalez et al. 2019; Ackerman 2021; Audi et al. 2023). This emerging interaction illustrates how nonnative species can generate new ecological relationships that impose selective pressures on both plants and pollinators (Figure 2e). For bees, novel phytochemicals may drive adaptations in detoxification or learning, while for plants, repeated visitation may influence the evolution of latex production.

Beyond direct effects, nonfloral foraging can alter plant‐herbivore dynamics. Bee activity on latex‐producing organs may deter herbivores by creating a chemically mediated “landscape of fear” (Laundre et al. 2010). Although bees do not actively defend plants, their buzzing and stinging can discourage herbivory, as demonstrated by African honeybee hives deterring elephant crop damage (Ngama et al. 2016). Such deterrence may indirectly benefit plants by reducing herbivore pressure and protecting seeds, potentially reinforcing selection for latex production (Figure 2f). Whether bees deter the same herbivores targeted by latex or a different set of consumers remains unknown, but either pathway suggests that bee foraging on plant exudates could have underappreciated consequences for plant defense and fitness.

In conclusion, taken together, this observation suggest that chemically mediated foraging by bees may play an overlooked role in shaping tropical ecological and evolutionary dynamics. By probing how bees recognize, utilize, and benefit from diverse plant chemicals, we gain insight into adaptive foraging strategies and the cascading effects of these behaviors on plant communities. We highlight the need for a more integrative approach to plant–bee ecology that considers chemical ecology, behavioral flexibility, and environmental context, especially in tropical landscapes where novel interactions are continually emerging.

## Author Contributions

**Luis Y. Santiago‐Rosario:** conceptualization (lead), data curation (lead), formal analysis (lead), funding acquisition (lead), investigation (lead), methodology (lead), project administration (lead), resources (lead), validation (lead), visualization (lead), writing – original draft (lead), writing – review and editing (lead). **Juan A. Santiago‐Rosario:** conceptualization (equal), investigation (equal), methodology (equal), resources (equal), writing – review and editing (equal). **Emilie C. Snell‐Rood:** conceptualization (equal), funding acquisition (supporting), resources (supporting), supervision (equal), validation (equal), writing – review and editing (equal).

## Funding

L.Y.S‐R. is funded by the National Science Foundation (award #2208922).

## Conflicts of Interest

The authors declare no conflicts of interest.

## Data Availability

No data was collected in this study.
